# Gender Dysphoria in 46,XX Persons with Adrenogenital Syndrome Raised as Females: An Addendum

**DOI:** 10.3389/fped.2014.00140

**Published:** 2015-01-06

**Authors:** Ricardo González, Barbara M. Ludwikowski

**Affiliations:** ^1^Pediatric Surgery and Urology, Auf der Bult Kinder- und Jugendkrankenhaus, Hannover, Germany

**Keywords:** congenital adrenal hyperplasia, gender identity, adrenogenital syndrome, feminizing genitoplasty, 21-hydr, 21-hydroxylase deficiency

In a recent opinion article (1), we incorrectly stated that to date no cases of gender dysphoria had been reported in 46,XX individuals with adrenogenital syndrome raised as females. Reflecting on this statement prompted this review.

In a review by Dessens et al. in 2005 (2), 13 of 250 (5.2%) cases of females with congenital adrenal hyperplasia (CAH) assigned the female gender role early in life later exhibited gender dysphoria of such severity that induced them to seek gender reassignment to male. Of interest, those cases originate from two publications (3, 4). A review of PubMed under the headings CAH and gender dysphoria yielded only one more article since 2005 (5).

This information is crucial to aid clinicians in counseling parents of affected newborns in gender assignment. According to the American Psychiatric Association, gender dysphoria is defined as a marked difference between expressed or experienced gender of a person and the gender that others would assign him or her, which continues for 6 months or longer (see www.dsm5.org).

It is well known that girls with salt wasting CAH tend to exhibit more tomboyish behavior and in adulthood may have less heterosexual preferences and may be less comfortable with their femininity. However, we were interested in finding out how many cases of XX individuals with CAH who actually decided to change their gender role after puberty have been reported, since this information is important for clinicians to advise parents in the neonatal period.

We thought it worthwhile analyzing the reported cases in detail. In Table 1 of Dessens et al.’s article, two cases are listed that were assigned the female gender at birth and in adulthood they identify themselves and live as males. Both of these patients were extracted from a 1996 report by Meyer-Bahlburg et al. (3). In this article, four XX individuals with CAH who were assigned the female gender in the first few weeks of life and in adulthood have a male gender identity and live as males. Two were compliant with glucocorticoid replacement therapy and two were not. It is impossible to know from the data presented if in the two compliant patients, the dose was sufficient to suppress production of androgenic precursors.

Another article reporting cases of gender dysphoria in CAH patients with XX phenotype is by Woelfle et al. (4). In a survey of German endocrinologists, they discovered 16 individuals with XX karyotype with “complete virilization” with an initially missed diagnosis who were raised as males. Six of them were reassigned to female in the first 19 months of life and they all maintain female gender role. Ten patients were diagnosed after 3 years of age. In seven patients, the male gender was maintained with apparently only one expressing doubts about gender identity. In three cases of late gender, reassignment to female was poorly tolerated in 1 of them.

Of course, with good neonatal care, Woelfle et al.’s series should have only historical interest in the developed world since all apparently male newborns with bilateral non-palpable testes should be emergently evaluated for possible CAH with karyotype and hormonal studies.

In a family from Saudi Arabia reported by Bin-Abbas et al. (5), three siblings with XX karyotype and CAH were raised as males. One exhibited problems with the male gender identity and change to female while the other two continued in their male gender role.

Zucker et al. (6) compared 31 patients with CAH with a control group. The authors concluded that the two groups “did not differ in degree of gender dysphoria in adulthood, although the probands showed more cross-gender role identification.” Three individuals who did not participate in the study lived as males. Two of them had been assigned the male gender at birth and one changed after puberty. Details of the hormonal status and compliance with the replacement therapy were not given.

Schober et al. (7) report includes one XX individual with salt wasting CAH whose history and hormonal status are not quite clear. Her gender identity was intersexual; sexual orientation was female with homosexual preferences. She had been assigned the male gender but identifies herself as female with homosexual preferences.

Gender role and gender dysphoria are a complex issue and are beyond the scope of this article. However, from a pediatric practitioner viewpoint, faced with newborn girls with CAH, the information provided in this review may prove helpful in counseling parents.

From this review we conclude that:

1. Gender dysphoria has been reported in XX individuals with CAH assigned the female gender at birth.
2. It is not clear if the cause of the gender dysphoria in the reported cases was due to exposure of the fetal brain to androgens or to poor compliance with glucocorticoid replacement therapy later in life.
3. In fact, severe gender dysphoria leading to gender change in adulthood was reported in only a few individuals diagnosed at birth and assigned the female gender, some of whom did not have glucocorticoid replacement.
4. Long term endocrine follow up of females with CAH is mandatory to prevent late virilization due to poor compliance with replacement steroid therapy.
5. The published data available cast serious doubts on the suggestion by Lee et al. (8) that severely virilized newborn females with CAH be raised as males.
6. All newborns with an apparently normal penis but non-palpable testes should be immediately evaluated for possible CAH and assigned the female gender if the diagnosis of 46,XX CAH is established.
7. We stand by the recommendations made in our earlier paper that genitoplasty should be done in first stage in the first year of life (1).

## Conflict of Interest Statement

The authors declare that the research was conducted in the absence of any commercial or financial relationships that could be construed as a potential conflict of interest.
